# Inactivation of the Catalytic Subunit of cAMP-Dependent Protein Kinase A Causes Delayed Appressorium Formation and Reduced Pathogenicity of *Colletotrichum gloeosporioides*

**DOI:** 10.1100/2012/545784

**Published:** 2012-05-01

**Authors:** Tri Puji Priyatno, Farah Diba Abu Bakar, Nurhaida Kamaruddin, Nor Muhammad Mahadi, Abdul Munir Abdul Murad

**Affiliations:** ^1^School of Biosciences and Biotechnology, Faculty of Science and Technology, Universiti Kebangsaan Malaysia, Selangor, 43600 Bangi, Malaysia; ^2^Malaysia Genome Institute, Jalan Bangi Lama, Selangor, Kajang 43000, Malaysia

## Abstract

The cyclic AMP- (cAMP-) dependent protein kinase A signaling pathway is one of the major signaling pathways responsible for regulation of the morphogenesis and pathogenesis of several pathogenic fungi. To evaluate the role of this pathway in the plant pathogenic fungus, *Colletotrichum gloeosporioides*, the gene encoding the catalytic subunit of cAMP-dependent protein kinase A, *CgPKAC*, was cloned, inactivated, and the mutant was analyzed. Analysis of the *Cgpkac* mutant generated via gene replacement showed that the mutants were able to form appressoria; however, their formation was delayed compared to the wild type. In addition, the mutant conidia underwent bipolar germination after appressoria formation, but no appressoria were generated from the second germ tube. The mutants also showed reduced ability to adhere to a hydrophobic surface and to degrade lipids localized in the appressoria. Based on the number of lesions produced during a pathogenicity test, the mutant's ability to cause disease in healthy mango fruits was reduced, which may be due to failure to penetrate into the fruit. These findings indicate that cAMP-dependent protein kinase A has an important role in regulating morphogenesis and is required for pathogenicity of *C. gloeosporioides*.

## 1. Introduction

The plant-pathogenic fungus *Colletotrichum gloeosporioides *(teleomorph: *Glomerella cingulata*) causes anthracnose diseases in a variety of crops in the subtropical and tropical regions [1]. Crops such as mango, avocado, papaya, coffee, and citrus have been infected with this fungus, which results in significant postharvest crop losses and limits the quality of the fruits produced for export. As demonstrated by several fungal pathogens, the pathogenicity of *Colletotrichum *depends on cellular morphogenesis. In response to physical and chemical signals, conidia of *Colletotrichum* species germinate on the host plants, form short germ tubes, and differentiate into specialized infection structures called appressoria [2]. The domed-shaped appressoria contain melanin layers, and in combination with the accumulation of glycerol, turgor pressure is generated inside the appressoria that eventually assists in the penetration of an infection peg into the host plant [3]. Upon entering the host, it will produce a network of internal hyphae to further penetrate and degrade plant cells.

The roles of fungal signal transduction pathways that relay information from the cells' surface receptors to the transcription machineries that lead to morphological changes and eventually enhance the pathogenicity of fungi during plant infection have been described for several phytopathogenic fungi. The major pathways that mediate the adjustment of intracellular activities in response to environmental changes include the cyclic AMP-dependent Protein Kinase A (cAMP-PKA) and MAP kinase signaling pathways [4–6]. The influence of the cAMP-PKA pathway in the development of morphogenesis and pathogenesis has been reported for various plant pathogenic fungi, including *Magnaporthe grisea*, *Ustilago maydis*, *C. trifolii*, and *C. lagenarium* [7–10]. Disruption of the gene encoding the catalytic subunit of cAMP-dependent protein kinase A, a downstream target of cAMP in these fungi, resulted in the alteration of morphogenesis and pathogenicity, albeit with varying degrees of defect. In *M. grisea*, deletion of the gene encoding this protein, *cpkA*, results in loss of the ability to produce normal appressoria, even in the presence of cAMP, and a complete loss of pathogenicity [11]. *M. grisea* CPKA is also involved in the regulation of lipid degradation, and this process produces glycerol that is required to generate appressorial turgor pressure [12]. In *U. maydis*, disruption of the gene that encodes the PKA catalytic subunit, *adr1*, resulted in constitutively filamentous growth and a nonpathogenic phenotype [8]. Similarly, *C. trifolii* mutants harboring a disruption in the catalytic subunit of PKA are nonpathogenic and unable to infect intact alfalfa (host) plants [10]. The mutants also showed a reduction in growth relative to the wild-type strain, and their conidiation pattern was altered. A *C. lagenarium* strain harboring a mutated PKA catalytic subunit, *cpk1*, was nonpathogenic on cucumber and germinated poorly, suggesting involvement of cAMP signaling in germination [9]. Germinated conidia of the mutants can form appressoria, but they are nonfunctional. In addition, c*pk1* mutants contained a larger number of lipid bodies compared to the wild-type strain, suggesting cAMP-mediated regulation of lipid metabolism for appressorium functionality as reported in *M. grisea* [9].

In *C. gloeosporioides*, Barhoom and Sharon [13] demonstrated that the cAMP is involved in the regulation of saprophytic germination. It was established that *C. gloeosporioides* exhibits two types of germination processes: saprophytic germination, which is induced by fermentable sugars, and pathogenic germination, which is triggered by chemical and physical plant surface signals. In contrast to saprophytic germination, the pathogenic germination is independent of the cAMP signaling pathway. However, similar to *M. grisea*, cAMP is required for appressoria formation in *C. gloeosporioides*. Addition of exogenous cAMP can induce appressoria formation even under conditions in which they do not normally form [13]. This indicates that cAMP is required for appressoria formation in *C. gloeosporioides* and could further regulate the conserved cAMP-PKA signaling pathway. However, whether this pathway in *C. gloeosporioides* is essential is not clear and requires further analysis.

Hence, to enhance the understanding of the role of the cAMP signaling pathway in the morphogenesis and pathogenicity of *C. gloeosporioides*, in this study, the gene encoding the catalytic subunit of protein kinase A was isolated, characterized, and inactivated by gene replacement. The mutants were then analyzed for their ability to germinate, undergo conidia-appressoria morphogenesis, and cause infection to the host.

## 2. Materials and Methods

### 2.1. Fungal and Culture Conditions

*C. gloeosporioides* PeuB was obtained from the stock culture collection of the School of Biosciences and Biotechnology, Universiti Kebangsaan Malaysia. The fungus was maintained by frequent subculturing on Potato Dextrose Broth (PDA: Difco, USA). Conidia, germinating conidia, appressoria, and mycelia were cultivated and harvested as described by Kamaruddin et al. [14].

### 2.2. Genomic DNA and RNA Isolation

Total DNA of *C. gloeosporioides* was isolated from mycelia using a method described by Oh et al. [15]. Total RNA of conidia, germinating conidia, and mycelia was extracted using TRI REAGENT solution (Molecular Research Center, USA), while RNA from the appressoria was extracted using TRIZOL solution in combination with mechanical cell disruption by glass beads [14]. Integrity and yield of the DNA and RNA were tested by agarose gel electrophoresis. Both DNA and RNA were stored at −20°C until further use.

### 2.3. Cloning of *CgPKAC* Gene and cDNA

The *CgPKAC *gene was isolated using a PCR-based strategy. The primers were designed based on the conserved regions of several fungal genes encoding the protein kinase A catalytic subunit. These primers are the CT1 (forward) 5′ ACA TTG GGA ACG GGT AGC TTC GGA AGA GTG 3′ (TLGTGSFGRV) and the CT2 (reverse) 5′ GTA GTC TGG CGT ACC GCA AAG CGT 3′ (TLCGTPDY). The PCR reaction was performed with one cycle at 94°C for 5 min, followed by 30 cycles at 94°C for 1 min, 65.7°C for 1 min and 72°C for 3 min, and one cycle of 20 min at 72°C. The purified PCR products were cloned into pGEM-T Easy vector (Promega, USA) and sequenced. Following sequencing, 5′ and 3′ rapid amplification of cDNA ends (RACE) PCR was performed using the SMART RACE cDNA amplification kit (Clontech, USA) following the protocol supplied by the manufacturer. Sequences of the primers used for the amplification, CGF and CGR, are shown in Table 1. The amplified products were cloned, and sequenced. To isolate the 5′ regulatory region of the gene, a DNA walking strategy using the DNA Walking SpeedUp kit (Seegene, Korea) was used. Three sequence-specific primers, designated as TSP1, TSP2, and TSP3 (Table 1), were used in the amplification reaction according to the manufacturer's instructions. The amplicons obtained were cloned, and sequenced. Subsequently, a 2.5 kb DNA fragment containing *CgPKAC,* along with its promoter and terminator, was amplified and cloned into pGEMT—Easy vector to generate pGEM-PKAC. For the isolation of the cDNA, RNA samples isolated from the mycelia were purified with Clean Up RNeasy (Qiagen, Germany) and treated with RNase-free DNase and used as template in a reverse transcriptase reaction (RT-PCR) using the Access RT-PCR kit (Promega, USA) following the manufacturer's protocol. The PCR amplicon was gel purified, cloned, and sequenced.

### 2.4. *CgPKAC* Gene Expression Analysis

Real-time quantitative polymerase chain reaction assays were conducted using the iCycler iQ Real-Time PCR Detection System (Bio-Rad, USA) and the iScript One-Step RT-PCR kit with SYBR Green (Bio-Rad, USA). RNA isolated from conidia, germinating conidia, mycelia, and appressoria was used as templates. Primers used to amplify a 75 bp *CgPKAC* DNA fragment (c-75F and c-75R) and 101 bp of the internal control, 18S rDNA DNA fragment (18SF2 and 18SR2), are shown in Table 1. All tubes were heated for 10 min at 50°C for cDNA synthesis followed by 5 min at 95°C for enzyme inactivation. Subsequently, each tube was subjected to a PCR amplification process of 94°C for 2 min followed by 44 cycles of 10 sec at 94°C and 30 sec at 60°C. Melt curve analysis was performed immediately after the amplification protocol under the following conditions: 1 min denaturation at 95°C, 1 min annealing at 55°C, and 80 cycles of 0.5°C increments (10 sec each) beginning at 55°C (data collection). All PCR products were electrophoresed on agarose gels (1%) to verify amplifications. All assays were carried out in triplicate and repeated with three independently isolated total RNAs. An appropriate control was also included (PCR reactions without DNA template). PCR fragments were cloned and sequenced to confirm that only the target sequence was amplified. Relative gene expression of *CgPKAC *was analyzed using 2^−ΔΔ*C*_*T*_^ method as described by Livak and Schmittgen [16]. Statistical analyses were performed using the SAS version 9.2 (SAS Institute Inc., USA) applying the one-way ANOVA test. Means were compared using the Duncan's multiple range test; when the *P* value was less than 0.05, the difference was regarded as statistically significant.

### 2.5. Construction of Gene-Replacement Vector, pN1389PKAC

For the construction of the *CgPKAC* replacement, the gene's 5′ region (−643 to +52) and 3′ region (+930 to +1306) were amplified from pGEM-PKAC. Both fragments were subcloned into vector pN1389 carrying a hygromycin expression resistance gene, resulting in the pN1389PKAC replacement plasmid. The 5′ region was amplified by PCR with PKACpN5-F and PKACpN5-R primers (Table 1) containing terminal *Kpn*I and *Bam*HI sites, while the 3′ region was amplified with primers PKACpN3-F and PKACpN3-R (Table 1), containing terminal *Sda*I and *Sph*I restriction sites, respectively. Both fragments were cloned into pGEM-T Easy, resulting in pGEM-PKAC5′ and pGEM-PKAC3′. Subsequently, Plasmid pN1389 was digested with *Kpn*I/*Bam*HI and ligated with 695 bp of the *CgPKAC* 5′ region fragment to produce plasmid pN1389PKAC5. Following that, pN1389PKAC5 was digested with *Sda*I/*Sph*I and ligated with 376 bp of the *CgPKAC* 3′ region fragment to produce the gene-replacement vector, pN1389-PKAC.

### 2.6. Transformation-Mediated Gene Replacement

Preparation of spheroplasts and transformation of *C. gloeosporioides* were performed according to methods described by Rodriguez and Redman [17]. Transformants were selected on regeneration medium containing hygromycin B (300 *μ*g mL^−1^) (Sigma, USA). Before transformation, pN1389-PKAC was linearized with the *Kpn*1 restriction endonuclease and precipitated with ethanol. Subsequently 20 *μ*g of DNA were transformed into *C. gloeosporioides *sphaeroplasts.

### 2.7. Genomic DNA and RNA Blot Analyses

DNA digestion, agarose gel fractionation, labeling of probes, and hybridization were performed according to the manufacturer's instructions and standard methods [18]. The 2.5 kb of full length *CgPKAC *or 695 bp of the 5′  *CgPKAC* fragment were labeled with [*α*-^32^P] dCTP using the Ready To Go DNA Labeling kit (-dCTP) (Amersham, USA). Hybridization was carried out with hybridization buffer [1 M Na_2_HPO_4_·2H_2_O, 1 M NaH_2_PO_4_, 0.5 M EDTA, 0.1% (w/v) SDS] at 65°C for 4 h for prehybridization and hybridized overnight after the labeled probes were added. The membrane was washed at 65°C with 2X SSC for 10 min followed by 2X SSC and 0.1% SDS, 1X SSC and 0.1% SDS, and finally 0.5X SSC and 0.1% SDS until the radioactivity signal was low. The washed blots were exposed to Fuji film for various times at −80°C.

### 2.8. Appressorium Induction on a Hydrophobic Hard Surface

Induction of appressorium was tested on a glass slide coated with rubber wax. A total of 50 *μ*L of wax (in chloroform) was spread on a glass slide with a cotton bud. Subsequently, 25 *μ*L of conidia suspension containing 10^5^ conidia mL^−1^ were applied on a glass slide. Appressorium formation was observed every hour for 8 h.

### 2.9. Assay for Appressorium Adhesion of *Cgpkac* Mutant

A test for the ability of the *Cgpkac* mutant appressoria to adhere to a hydrophobic hard surface was carried out using a protocol as described by Lapp and Skoropad [19]. Briefly, 20 *μ*L of conidial suspension containing 10^4^ conidia mL^−1^ were induced to form appressoria onto a hydrophobic glass slide coated with rubber leaf wax. After incubation for 12 h, the glass slide was washed with sterile distilled water to remove ungerminated conidia and germ tubes and left to dry. Subsequently, appressoria that were attached to the glass slide were treated with 50 *μ*L of 4% sodium hydroxide and incubated for 2 h at ambient temperature. Sterilized distilled water was used to treat controls. After incubation, sodium hydroxide solution was pipetted and transferred into a microtube, while appressoria remaining on the glass slide were rinsed with 1 mL of distilled water. Sodium hydroxide solution and 1 mL of distilled water were then centrifuged to collect any appressoria that had been removed. The number of appressoria that were attached to the glass slide was counted under a light microscope (Olympus, Germany).

### 2.10. Detection of Lipid Bodies in Appressoria

Conidia of *C. gloeosporioides* were harvested from seven-day-old cultures grown on PDA. Conidia were suspended in 10 *μ*g mL^−1^ of carpropamid solution (Sigma-Aldrich, USA) and incubated on glass slides coated with rubber leaf wax for 24 h. Non-melanized appressoria were stained with a Nile Red (Fluka, Germany) solution at 2.5 *μ*g mL^−1^ for 10 min in the dark [20]. Nile Red was prepared by mixing with acetone (1 mg mL^−1^) and diluted at 1 : 100 in phosphate buffer saline, pH 7. To facilitate diffusion of Nile Red into nonmelanized appressoria, polyvinylpyrrolidone was added to the buffer at a concentration of 20 mg mL^−1^ [12]. Images were captured with a Leica phase-contrast microscope. Fluorescence intensity was calculated using a 1D-multi analysis tool from AlphaEaseFC Software provided with the AlphaImager Gel Imaging system (Alphainnotech, UK).

### 2.11. Virulence Assay

A test for pathogenicity was performed as described by Kim et al. [21]. Mature green mangos were infected with conidia of *C. gloeosporioides.* Both unwounded and wounded mango fruits were inoculated. Before inoculation, fruits were surface-sterilized with 70% ethanol and left to dry at room temperature. Fruits were wounded with a sterilized pin stabbed five times at the localized areas. A total of 0.5 mL of conidial suspensions at 2 × 10^4^ conidia mL^−1^ was applied on to the surface of unwounded fruits by spraying the inoculum with a spray gun (Preval, USA), while wounded fruits were inoculated with 20 *μ*L of conidial suspension. Mangos were arranged in a moistened plastic tray and incubated at 30°C for two weeks to observe disease symptoms. The number of lesions was observed daily.

## 3. Results

### 3.1. Cloning and Characterization of *C. gloeosporioides  CgPKAC*

A PCR-based screen with primers based on conserved regions of the *PKAC *gene of several fungi was used to amplify 0.5 kb of the *PKAC* gene of *C. gloeosporioides* (*CgPKAC*). Primers yielded a single PCR fragment of about 500 bp. The predicted amino acid sequence of this fragment showed a high homology to other fungal PKACs; therefore, this fragment was used as template to isolate the rest of the gene sequence using RACE (Rapid Amplification of cDNA Ends)-PCR and a DNA Walking strategy. Based on 500 bp of the partial *CgPKAC* sequence, two primers were designed for RACE-PCR. Primer CGF and CGR in combination with universal primer supplied by a cDNA SMART RACE kit were used to amplify the 5′ and 3′ region of *CgPKAC*, respectively. Primer CGF and a universal primer yielded a PCR fragment of about 1.1 kb, while primer CGR and a universal primer yielded a 1.3 kb fragment. The sequences of both fragments were overlapped with known sequences used to generate a primer. Subsequently, the 1.1 kb sequence from the 5′ RACE-PCR was used to design three primers to amplify the upstream region of the *CgPKAC* gene using the DNA Walking kit. In the final step of DNA Walking, a 1.0 kb upstream fragment of *CgPKAC* was amplified, cloned, and sequenced.

**Figure 1:**
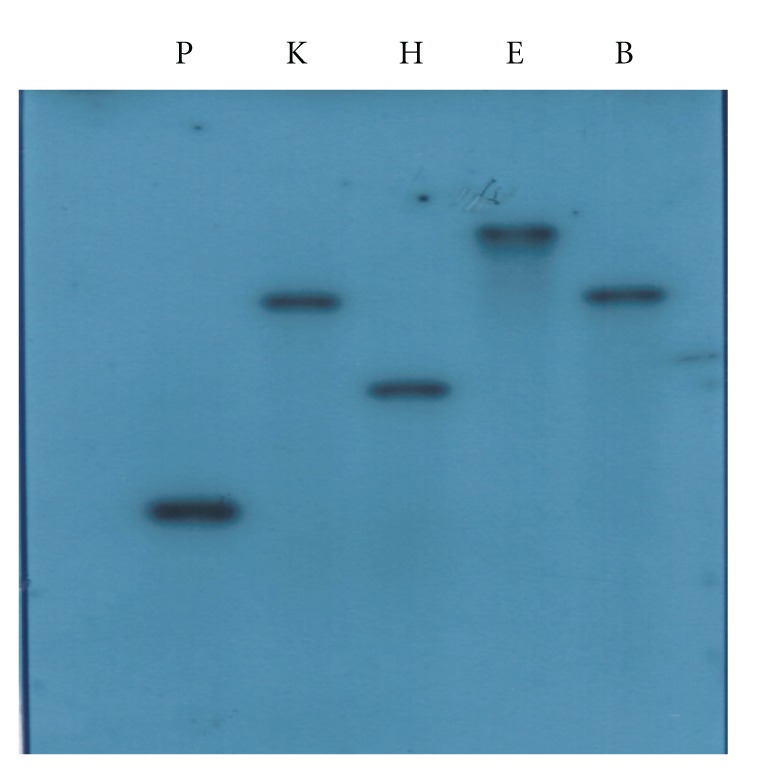
Southern blot analysis of* CgPKAC* using genomic DNA of wild-type *C. gloeosporioides*. Genomic DNA (8 *μ*g/Lane) of *C. gloeosporioides* wild-type wtrain was digested with *Pst*1 (P), *Kpn*1 (K), *Hin*dIII (H), *Eco*R1 (E) and *Bam*H1 (B). The blot was probed with a 695 bp of *CgPKAC* fragment.

Based on the sequence information obtained via RACE-PCR and DNA Walking, a DNA fragment of 2597 bp containing the *CgPKAC* open reading frame (ORF) along with 731 bp of its 5′ upstream region and 184 bp of the 3′ region was obtained. *CgPKAC* consists of a 1683 bp open reading frame, and by comparing the gene sequence with its corresponding cDNA, three introns of 59 bp, 69 bp, and 52 bp were identified. The cDNA encodes for a 500 amino acid protein with a putative molecular mass of 56 kDa. The size of the *CgPKAC* ORF was approximately similar to the PKA catalytic genes of *M. grisea*, 1620 bp [11], *U. maydis*, 1197 bp [8], *C. trifolii*, 1593 bp [10], and *C. albicans*, 1329 bp [23]. The deduced amino acid sequence of *CgPKAC* also shares significant homology with those of the PKA catalytic genes of *C. lagenarium *(84% identity), *C. trifolii *(82%), *A. niger *(83%), and *Metarhizium anisopliae *(69%). Putative TATA and fungal CAAT boxes were found upstream from the start codon at positions −22 and −258, respectively. The *CgPKAC* gene sequence has been submitted to GenBank with the accession number DQ812968. Southern blot analysis with genomic DNAs digested with *Bam*H1, *Eco*R1, *Hin*dIII, *Kpn*I, and *Pst*I indicates that *CgPKAC* is a single-copy gene in the genome of *C. gloeosporioides* (Figure 1).

### 3.2. Expression of *CgPKAC* in Various Developmental Stages

**Figure 2:**
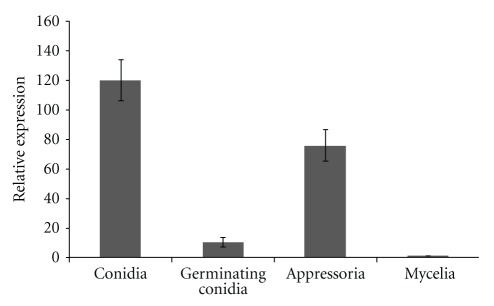
Expression level of *CgPKAC* in different morphological cells; conidia, germinating conidia, appressoria, and mycelia. 18S rDNA was used as a reference gene and the expression of *CgPKAC* in different morphological cells was compared to the level of its expression in the reference sample (mycelia). Statistically significant differences in *CgPKAC* expression levels between conidia, germinating conidia, appressorian, and mycelia were tested with an ANOVA analysis (*P* < 0.05).

A real-time quantitative RT-PCR assay was performed to quantitate *CgPKAC* gene expression in different morphological cells using SYBR green as a measurement of PCR product formation. How *CgPKAC* expression is regulated during conidia-appressoria morphogenesis is still unknown. In this work, the expression of the *CgPKAC* in different cDNA samples was compared to the level of its expression in the reference sample, which is the cDNA from mycelia. As such, expression of the catalytic subunit gene in the mycelia cDNA sample was assigned the value of 1.0. The amplification efficiencies of *CgPKAC* and 18S rDNA were relatively equal, thus allowing the use of the comparative Ct method for relative quantification as described by Livak and Schmittgen [16]. Results of this work indicate that the expression of *CgPKAC* is developmentally regulated at least at the level of transcription. Relative expression of *CgPKAC* was found highest in conidia with 120-fold, appressoria with 76-fold, and germinating conidia with 10-fold as compared to mycelia (reference sample) (Figure 2).

### 3.3. Inactivation of *CgPKAC*

**Figure 3:**
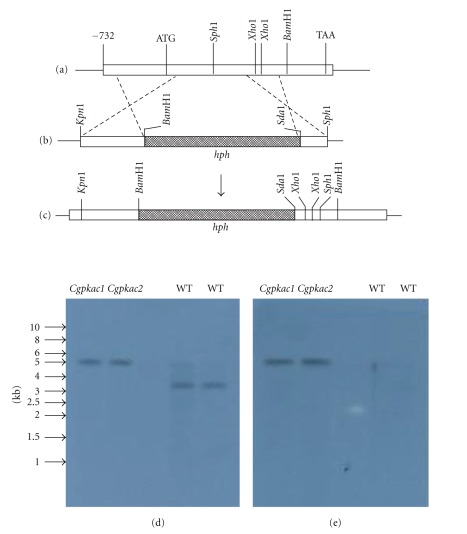
Schematic presentation of *CgPKAC* gene disruption and DNA blot analysis of *CgPKAC* gene replacement. (a) Predicted restriction map of the *CgPKAC* locus in the *C. gloeosporioides* genome. (b) Gene replacement vector pN-CPKA. The dotted line between (a) and (b) represents the crossing over between two homologous regions that may occur during *CgPKAC *gene disruption. (c) Predicted restriction map of the *Cgpkac* deleted allele. (d) Genomic DNA was digested with *Xho*1 and probed with a 695 bp of 5′*CgPKAC* fragment. (e) Genomic DNA was digested with *Xho*1 and probed with a 1.1 kb of *hph* fragment. In the wild type, a single 3.5 kb DNA fragment was detected whilst for the transformants *Cgpkac1* and *Cgpkac2*, a 5.8 kb band was identified when probed with *CgPKAC*. Only the *Cgpkac1 and Cgpkac2* transformants showed positive signals when probed with the *hph* gene.

**Figure 4:**
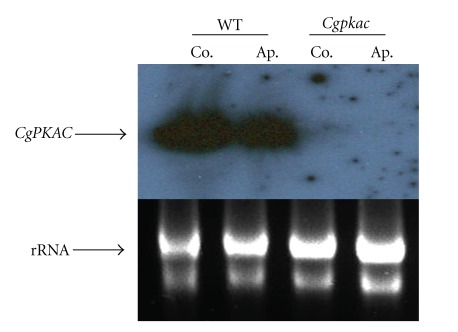
Northern blot analysis of total RNA obtained from conidia (Co.) and appressoria (Ap.) of the wild type (WT) and *Cgpkac* mutant (*Cgpkac*). The RNA was hybridized with 2.5 kb *CgPKAC*.

DNA-mediated gene replacement was performed to assess the role *CgPKAC* in *C. gloeosporioides* with the gene deletion construct shown in Figure 3. *C. gloeosporioides* sphaeroplasts were transformed with the pN1389-PKAC gene replacement plasmid that was linearized with *Kpn*1. From seven transformants that were able to grow on regeneration medium containing 300 *μ*g hygromycin, only three transformants showed mitotic stability on PDA-hygromycin medium. To confirm that the integration of the hygromycin-resistant gene cassette occurred at the *CgPKAC* gene in the genome, transformants were initially screened by PCR and subsequently confirmed by Southern blot analysis. Figure 3 shows the result of the Southern blot analysis of DNA from the wild-type and mutant strains that was digested with *Xho*1 and probed with the 1.1 kb hygromycin (*hph*) fragment and the 2.5 kb *CgPKAC* fragment. Two transformants, *Cgpkac1* and *Cgpkac2*, produced a positive signal when probed with the *hph *fragment, which indicated that the hygromycin gene deletion cassette was integrated into their genome following transformation. Hybridization with the *CgPKAC* gene resulted in the formation of a 5.8 kb DNA fragment and thus confirmed the integration of the gene deletion cassette into the target gene (Figure 3). To confirm total deletion of *CgPKAC*, the presence of its transcript in one of the mutants was examined by Northern blot analysis. Total RNA from the wild-type strain and the *Cgpkac* mutant was obtained from conidia of a 7-day-old culture grown on PDA and 7 h appressoria formed on a glass plate layered with rubber leaf wax. The RNA was hybridized with 2.5 kb of *CgPKAC*. The results showed that there was no *CgPKAC* transcript detected in the mutant as compared to the wild type indicating that the gene had been completely inactivated in the mutant (Figure 4).

### 3.4. Inactivation of *CgPKAC* Caused Both a Delay in Appressorium Formation and Bipolar Germination

**Figure 5:**
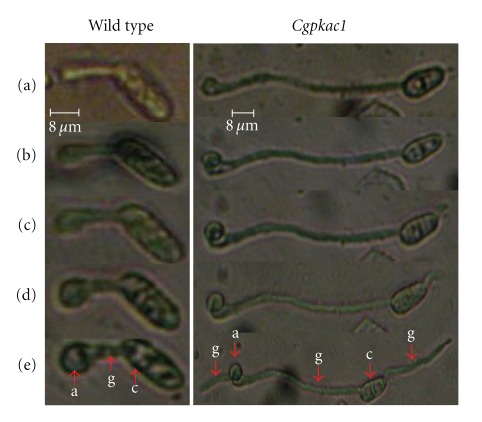
Progressive phases of *C. gloeosporioides* mutant *Cgpkac1 *and wild-type strain during conidial germination and appressorium formation. The image was captured using an Olympus phase contrast microscope (400x magnification) and a Nikon digital camera. Appressorium formation of 4-hour-old germlings on hydrophobic glass slide coated with rubber wax was observed at (a) 0 min; (b) 30 min; (c) 60 min; (d) 90 min; (e) 180 min. (a: appressorium; c: conidium; g: germ tube).

**Figure 6:**
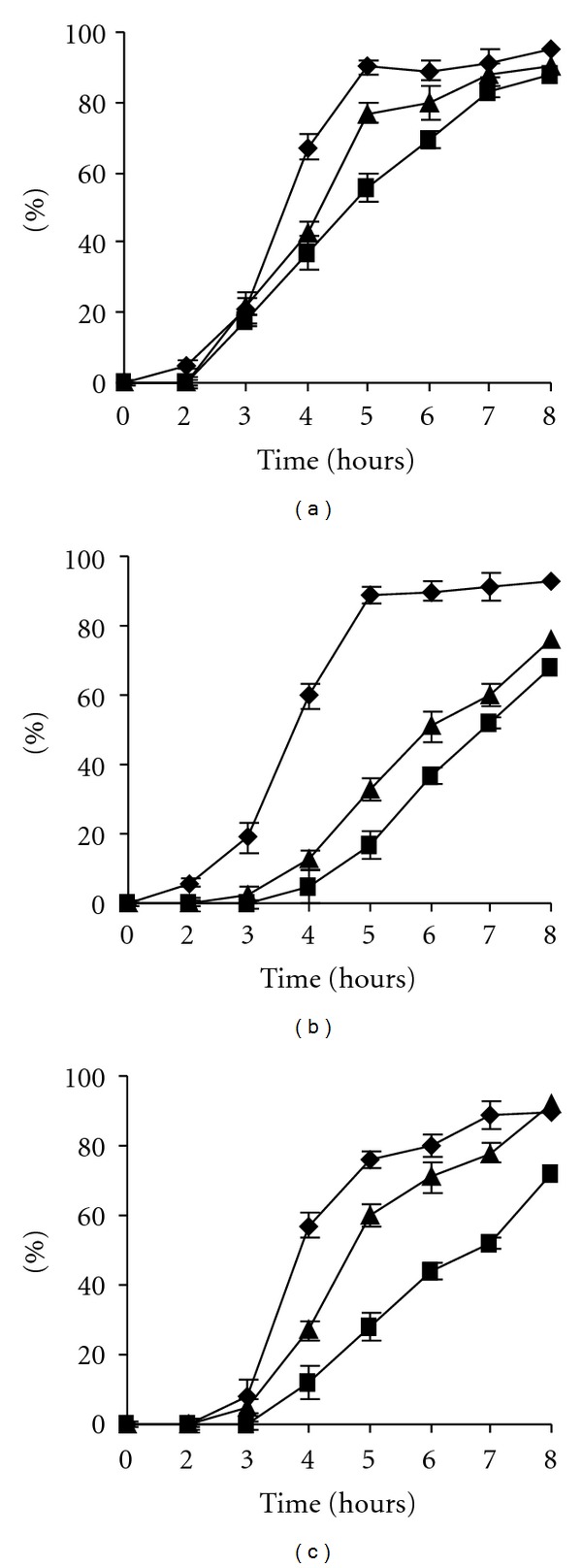
Germination and appressorium formation of the wild type (a), *Cgpkac1* (b), and *Cgpkac2* mutants (c) of *C. gloeosporioides*. Germination (*◆*), hooking (▴), and appressorium formation (▪).

**Figure 7:**
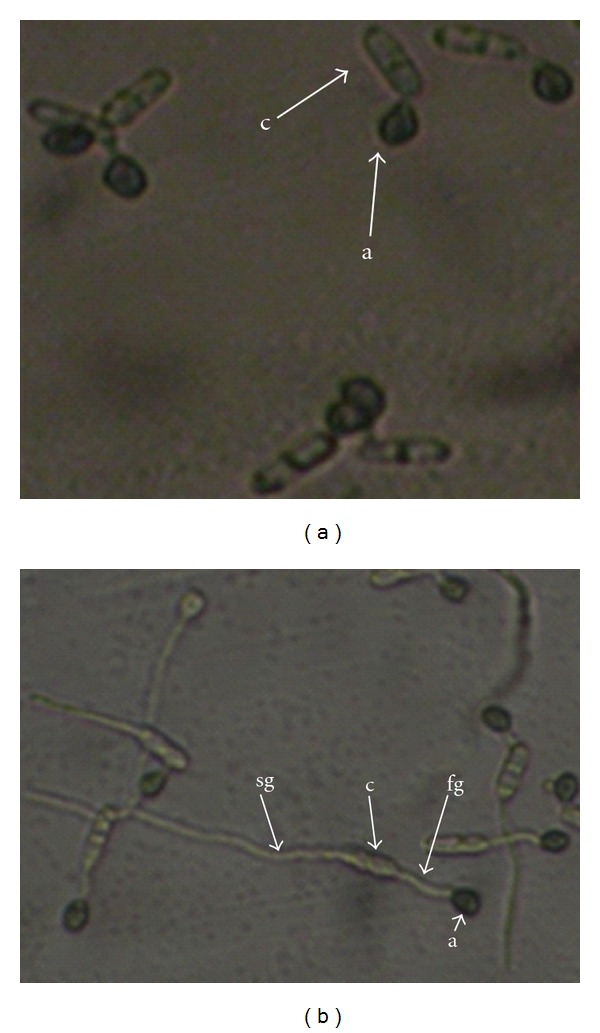
The *C. gloeosporioides* wild type (A) strain and *Cgpkac1* mutant (B) displaying appressoria and bidirectional germ tubes. The image was captured with an Olympus phase contrast microscope (200x magnification) and a Nikon digital camera. (a: appressorium; c: conidium; fg: first germ tube; sg: second germ tube).

Observation of morphogenesis of the *Cgpkac* mutants indicated no reduction in conidiation and growth relative to the wild-type strain on rich PDA medium. Conidia of *Cgpkac* mutants had a normal morphology and germination rate. Mutant conidia also showed no defects in germ tube hooking when they were exposed to the hydrophobic surface of glass slides coated with rubber leaf wax. *Cgpkac *mutants were able to form appressoria; however, morphogenetic development of these mutants was different when compared to the wild type.  Figure 5 shows the differences in appressoria development of the mutant and wild-type strains. Mutants' appressoria were formed at the tip of a long germ tube, while the wild-type strain formed sessile appressoria. The percentage of sessile appressoria formed by *Cgpka1 *and *Cgpka2 *mutants were only 17.1 ± 4.2% and 12.7 ± 7.6%, respectively, as compared to 89.2 ± 5.3% of sessile appressoria formed by the wild-type strain. Formation of sessile appressoria was an indicator showing fast response of germlings to external stimuli leading to appressorium formation [24]. This indicates that inactivation of cAMP-dependent protein kinase A delayed appressorium formation in *C. gloeosporioides*. Initiation of appressorium formation was delayed at least 3 h in the mutant compared to the wild-type strain. After 8 h, more than 80% of the wild-type conidia produced appressoria, while less than 60% of the *Cgpkac* mutant conidia produced appressoria (Figure 6). Nevertheless, after more than 12 h of induction, the percentage of appressoria produced from the mutant conidia was similar to the wild type. In addition, mutant conidia could also germinate to form a second germ tube, which was formed in the opposite direction of the first germ tube (Figure 7). The emergence of the second germ tube from the mutant's conidia only appeared after complete appressorium was generated at the tip of the first germ tube. However, no appressorium was generated from the second germ tube.

### 3.5. *Cgpkac* Appressoria Exhibit Impaired Ability to Adhere to Hydrophobic Surface

Appressoria adhere tightly to surfaces and their primary role is to secure penetration into the host. In order to determine whether mutant appressoria formed at the tip of their extended germ tubes adhere tightly onto hydrophobic surfaces, 12 h-old appressoria of the *Cgpkac* mutant and wild-type strains were treated with 4% sodium hydroxide and incubated for 2 h at room temperature [19]. The results showed that appressoria of *Cgpkac* mutants demonstrated a reduced ability to adhere to the hydrophobic surface as compared to wild-type appressoria. Approximately 71.8 ± 11.2% and 68.6 ± 9.2% appressoria of *Cgpkac1* and *Cgpkac2* were removed from the glass slide after treatment with 4% for 2 h, while only 39.2 ± 8.9% of the wild-type appressoria were removed. This indicates that the mutant appressoria failed to adhere tightly to the hydrophobic surface. These results suggest that the cAMP-PKA signaling cascade could be responsible for the regulation of genes required for adhesion of appressoria onto host surfaces.

### 3.6. Degradation of Lipid Bodies in Appressoria

**Figure 8:**
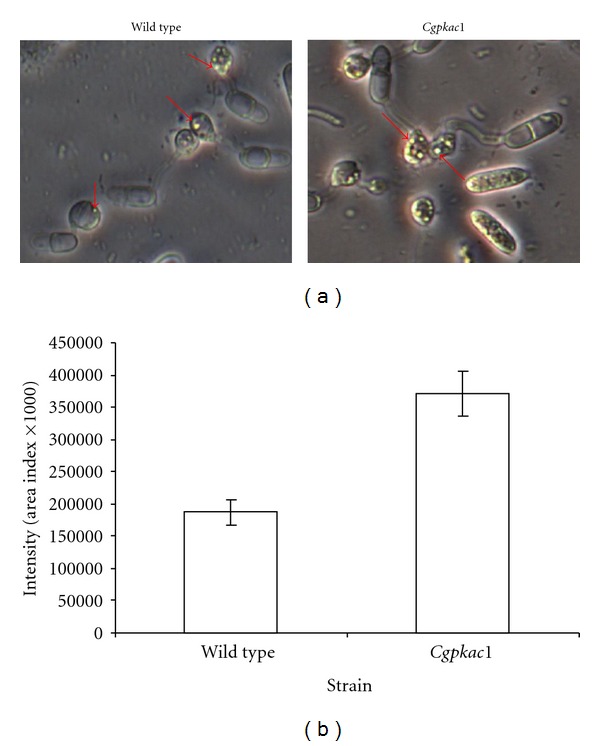
Cellular distribution of lipid droplets in *C. gloeosporioides *wild type and the *Cgpkac1* mutant. (a) The presence of Nile-Red-stained lipid appressoria was observed with a Leica phase contrast microscope (400x magnification). Arrows indicate fluorescent lipid stained with 10 *μ*g of Nile Red. (b) Area index indicating lipid content in appressoria of the wild-type strain and *Cgpkac1* mutant was based on analysis using AlphaEaseFC Software.

To detect lipid bodies in appressoria, nonmelanized appressoria were stained with Nile Red [20]. Since incorporation of Nile Red into lipid bodies is more efficient in non-melanized appressoria as compared to intact appressoria, the formation of non-melanized appressoria was induced by treating germinating conidia with capropamid on glass slides coated with rubber leaf wax [12]. The appressoria treated with capropamid are completely colorless and transparent, and easily differentiated from normal appressoria. Capropamid inhibits scytalone dehydratase, an enzyme involved in fungal melanin biosynthesis [22]. When stained with Nile Red, microscopic analysis revealed that the number of lipid bodies inside the appressoria differed between the wild type and the mutants. The intensity of lipid stained with Nile red was detected twofold higher in the mutant as compared to the wild type (Figure 8). Appressoria of the *Cgpkac1* mutant retained lipid bodies even after 24 h. At this point, the presence of lipid bodies in the appressoria of the wild type strain was still detected; however, significant reduction was observed in the wild type as compared to the mutant strain. This result may be indicative of the involvement of the cAMP-PKA signaling pathway in lipid degradation, which in turn could generate appressorial turgor pressure in *C. gloeosporioides*, as observed in *M. grisea* and *C. lagenarium* [9, 12].

### 3.7. *CgPKAC* Is Required for *C. gloeosporioides* Pathogenicity

**Figure 9:**
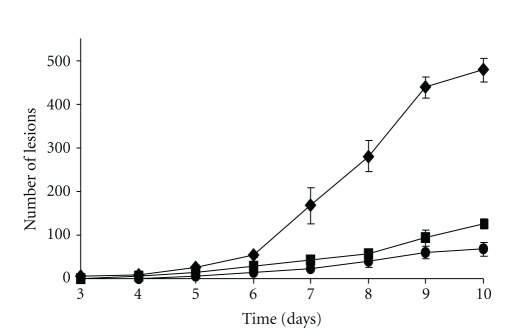
Disease severity of unwounded mango inoculated with the wild type. (*◆*), *Cgpkac1* (▪), and *Cgpkac2 *(*⚫*) mutants.

**Figure 10:**
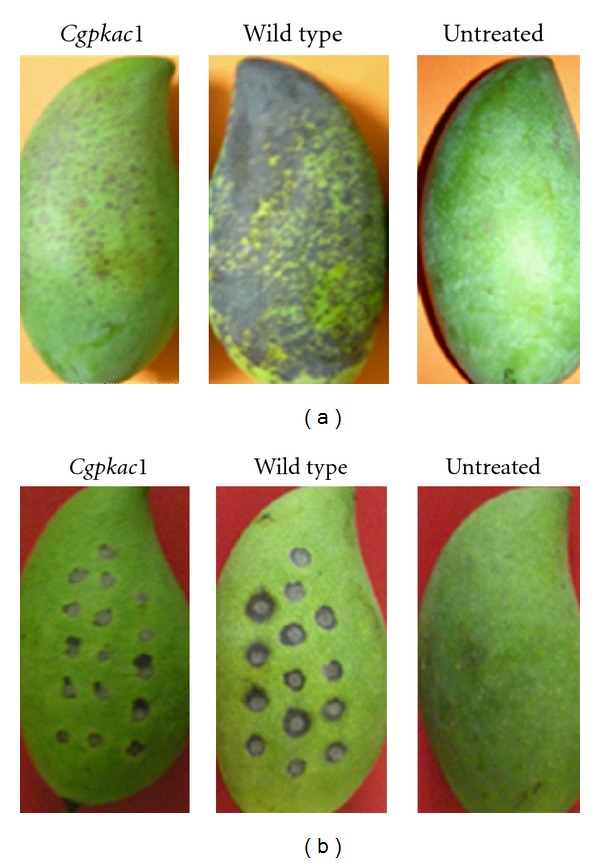
Pathogenicity assays of *C. gloeosporioides *wild-type strain and *Cgpkac1 *mutant on unwounded (a) and wounded (b) mango fruits. A 0.5 mL conidia suspension containing 10^5^ conidia mL^−1^ was sprayed onto unwounded fruits, while wounded fruits were inoculated with 20 *μ*L of conidial suspension. Untreated fruits were treated with sterile distilled water. Anthracnose symptoms were observed daily for ten days.

The most significant phenotype of the *Cgpkac* mutants was their inability to infect intact mango fruits. After five days, lesions were observed in the wild-type-inoculated fruits. Acervuli and abundant mycelial growth were observed in the lesions caused by the wild-type strain. In contrast, very small lesions in low abundance were observed on the mango fruits infected with the mutant (Figures 9 and 10). To test whether the failure to infect hosts was due to impairment in penetration, the hosts were wounded and infected with the wild-type strain and the *Cgpkac* mutant. After five days, both the wild-type strain and the mutant showed the ability to colonize the host cells; however, the *Cgpkac* mutant produced smaller lesions as compared to the wild-type strain, indicating that the mutant was nonaggressive (Figure 10).

 The fact that these *Cgpkac* mutants could form appressoria and colonize wounded fruits suggested that the loss of pathogenicity was not due to the impairment colonization or appressoria formation, but was most likely due to a failure in appressoria penetration. Sessile appressoria that are formed often in the wild-type strain are thought to be more effective at penetrating host surfaces as compared to the mutant appressoria. Similar results have been reported for *M. grisea* [11] and *C. trifolii PKAC* mutants [10].

## 4. Discussion

cAMP-PKA signaling regulates morphogenesis and virulence in a wide variety of fungi, including plant and animal fungal pathogens. Although this signaling cascade is highly conserved among fungi [5], disruption of the cAMP signaling cascade has resulted in various effects. For example, this pathway is required for filamentous growth in the human fungal pathogen *C. albicans*, and mutation of major proteins in the pathway, including adenylate cyclase, Cdc35, and catalytic subunits of protein kinase A, Tpk1 and Tpk2, inhibit filamentous growth [25]. However, in the plant pathogen, *U. maydis*, this pathway is required for budding growth, since increased expression of the adenylate cyclase, Uac1, or the catalytic subunit of protein kinase A, Adr1, suppresses filamentous growth; in contrast, deletion of *adr1* procures the opposite effect [26]. In appressorium-producing fungal plant pathogens, this pathway is generally required for pathogenicity and appressorium morphogenesis. However, inactivation of this pathway resulted in different degrees of impairment in appressorium formation. For example, deletion of the gene encoding the catalytic subunit of protein kinase A in *M. grisea*,* cpkA, *resulted in severely delayed appressorium formation [27], while mutation of the same gene in *C. trifolii* resulted in no differences in the development of appressoria as compared to the wild type [10]. In contrast, inactivation of this protein in *C. lagenarium* generates mutants that germinated poorly on an inductive surface even after prolonged incubation [9]. However, at lower conidia density, the mutants formed appressoria, but were nonfunctional. Due to these differences, it is important to understand the role of this signaling pathway in other fungal species, since this will enhance our knowledge of the contribution of this pathway in fungal morphogenesis and pathogenesis.

Disruption of *CgPKAC* resulted in mutants that showed normal growth and conidiation on rich media. The morphologies of the conidia and mycelia of the mutants were the same as the wild-type strain. However, when the mutant conidia were induced for appressoria morphogenesis, the formation of appressoria in the mutants was delayed when compared to the wild-type strain. When induced, both mutant and the wild-type conidia germinated at almost the same rate; however, the conidia of *Cgpkac* mutants formed long germ tubes before differentiating into appressoria. The initiation of appressorium formation in the mutant was approximately 3 h after induction. In contrast, the wild-type appressoria was generated from short germ tubes and started to form sessile appressoria less than 1 h after induction. However, after prolonged induction (more than 12 h of induction), the percentage of appressoria produced from the mutant conidia was similar to the wild type. This observation suggests that the cAMP-PKA signaling cascade is important to relay signals for conidium-appressorium differentiation in *C. gloeosporioides*, since inactivation of this pathway delayed appressoria morphogenesis. This observation also indicates that there is/are other signal transduction pathway/s in *C. gloeosporioides* that can transfer morphogenetic signals in response to plant wax and hard surfaces, since inactivation of the cAMP-PKA signaling pathway did not completely shut off conidium-appressorium morphogenesis. Kim et al. [28] showed that deletion of *C. gloeosporioides* mitogen-activated protein (MAP) kinase kinase resulted in mutants that were unable to form appressoria, suggesting that the MAP kinase pathway is one of the pathways required for transferring morphogenetic signals and is presumably the more dominant pathway as compared to the cAMP-PKA signaling pathway.

Besides delayed appressoria formation, the conidia of *Cgpkac* mutants underwent bipolar germination upon completion of the first appressoria formation. The second germ tube was produced from the parent conidia in the opposite direction of the first germ tube. However, no appressoria were generated from the second germ tube even after prolonged incubation. This result indicates that the cAMP-PKA pathway is not only important for the regulation of appressoria formation, but also, to some extent, represses saprophytic conidial germination during pathogenic growth. In the absence of active PKA, the conidia are activated to form another germ tube, which is saprophytic in nature, after the formation of the appressoria. Cells might recognize that the appressoria formed were nonfunctional, as they were unable to penetrate hosts leading to colonization, and decided to undergo another round of germination to produce saprophytic mycelia. However, this event does not occur in the wild-type strain, suggesting that the cAMP-PKA signaling cascade might activate a repressor protein that inhibits the germination of saprophytic germ tubes from the parent conidia after appressoria were generated. This observation is also in agreement with the fact that *CgPKAC* is highly expressed in the conidia and appressoria of *C. gloeosporioides*. This reflects the importance of the activities executed by this protein to relay information in these two cell morphologies, which is required for their survival on host cells.

Even though appressoria of *Cgpkac* mutants were formed at the tip of a long germ tube, they formed normal melanin as in the wild type. However, the ability of the mutant appressoria to adhere to hydrophobic surfaces was reduced when compared to the wild-type strain. This observation suggests that the cAMP-PKA signaling cascade could play an important role in regulating appressoria-specific adhesion factors. Inactivation of protein kinase A may limit the signals required to activate the expression of genes that encode for these appressoria proteins. The regulation of adhesion factors by the cAMP-PKA signaling pathway has been reported in the human pathogen *C. albicans*, whereby it was shown that this pathway regulates the expression of several hyphae-specific adhesins, such as the cell surface adhesins, Hwp1, and Als3 [29].

To penetrate host cells, the appressoria of fungal pathogens, such as *M. grisea*, *C. lagenarium*, and *C trifolii*, produce penetration pegs that can pierce through the cuticle of plant cells via mechanical forces. The mechanical force is generated by enormous cellular turgor pressure via accumulation of a high concentration of glycerol in melanized appressoria, which allows the fungus to send a narrow penetration peg through the host cuticle [30, 31]. Thines et al. [12] reported that *M. grisea* conidia accumulated lipids, glycogen, and the disaccharides trehalose, as the predominant storage products and metabolism of these storage products contributed to glycerol formation and eventually to the cellular turgor pressure of the appressorium. During appressorium maturation of *M. grisea*, trehalose, glycogen, and lipid have all been found to be degraded rapidly [12, 32]. The regulation of lipases and glycogen-degrading enzymes by PKA phosphorylation has been described in mammalian systems [33], and PKA is a central regulator of carbohydrate mobilization in yeast [34]. In *M. grisea*, PKA is involved in the regulation of lipid degradation to produce glycerol, which contributes to appressorial turgor pressure [12]. Deletion of the *M. grisea cpkA* gene resulted in retardation of lipid and glycogen degradation, while deletion of the *SUM1* gene, encoding the regulatory subunit of protein kinase A, resulted in rapid degradation of lipid and glycogen [35]. Similarly, deletion of *CPK1 *in *C. lagenarium* inhibited degradation of lipid bodies [9]. In this work, we observed that *C. gloeosporioides Cgpkac* mutant appressoria contain more lipid bodies as compared to the wild type. This indicates that the mutants were unable to degrade lipid as efficiently as the wild type and that the cAMP-PKA signaling cascade is important in regulating proteins required for lipid degradation in *C. gloeosporioides*.

The reduction in the ability of the *Cgpkac *mutant appressoria to adhere to hydrophobic surfaces and to degrade lipids for glycerol accumulation correlated with the results of the virulence assays, whereby infection of unwounded mango fruits with *Cgpkac *mutant conidia produced nonintact lesions compared to the wild-type strain. However, *Cgpkac *mutants of *C. gloeosporioides* were able to colonize host tissues following artificial wounding. These observations suggest that the loss of pathogenicity of the mutant is most probably due to a failure in appressoria penetration. These defects could be due to the inability of the mutant appressoria to adhere tightly to the host surface and to generate enough turgor pressure inside the cells to produce a penetrative penetration peg. As reported in other phytopathogenic fungi, such as *M. grisea* and *C. lagenarium*, loss of pathogenicity was observed for deletion mutants of the PKA subunit catalytic gene and was attributed to nonfunctional appressoria [9, 35].

In summary, we conclude that in *C. gloeosporioides*, the cAMP-PKA signaling pathway is not essential for conidia-appressorium morphogenesis, since the mutants were able to form appressorium, even though their formation was delayed. The delay in appressorium formation suggests that this pathway is required for transfer of some of the morphogenetic signals, that this most likely occurs in parallel with other signaling pathway/s, and that the defects in the cAMP-PKA signaling pathway will be rescued by the alternative pathway/s. However, this pathway is critical for the function of the appressorium. Blocking of cAMP signaling via deletion of the PKA catalytic subunit reduces the ability of the appressoria to adhere to hydrophobic surfaces, most probably due to the absence of certain adhesion molecules and leads to the failure to generate enough turgor pressure, which is due to the inability to convert lipid bodies into glycerol. These defects resulted in appressoria that were unable to generate penetration pegs to penetrate through the host surface and led to a loss in pathogenicity. The results of this study also suggest that the cAMP-PKA signaling pathway may regulate effects through a repressor protein that inhibits the germination of saprophytic germ tubes following a parasitic mode of growth. This regulation is important for fungal cells' survival upon failure of their parasitic machineries and their need to then switch to an alternative growth mode to survive and proliferate.

## Figures and Tables

**Table 1:** Oligonucleotide primers used in this study.

Name	Description	Sequence
c-75F	Forward primer to amplify 75 bp *CgPKAC* partial gene during real-time RT-PCR	5′-GGTCTCATAAATCATGTTTGCACTG-3′
c-75R	Reverse primer to amplify 75 bp *CgPKAC* partial gene during real-time RT-PCR	5′-CGTCATTGCTTTCCTATCCAT-3′
18SF2	Forward primer to amplify 101 bp 18S rDNA partial gene during real-time RT-PCR	5′-CAGCGAAATGCGATAAGTAATG-3′
18SR2	Reverse primer to amplify 101 bp 18S rDNA partial gene during real-time RT-PCR	5′-GCAGAGCTTGAGGGTTGAAAT-3′
CGF	Primer used for 5′-RACE PCR amplification	5′-GTCCGACAGACGAAGGGGAAATAC-3′
CGR	Primer used for 3′-RACE PCR amplification	5′-CCACGGATTTGTTGTAGCCCTTGT-3′
TSP1	Template-specific primer used in DNA walking for *CgPKAC* regulatory region amplification	5′-GCAGCGAGAAGAGTTTCACCAC-3′
TSP2	Template-specific primer used in DNA walking for *CgPKAC* regulatory region amplification	5′-GTATTTCCCCTTCGTCTGTCGG-3′
TSP3	Template-specific primer used in DNA walking for *CgPKAC* regulatory region amplification	5′-GATTAGGAGGATGGATGGTGAC-3′
PKACpN5-F	Forward primer to amplify 695 bp 5′ region of *CgPKAC* for construction into hygromycin cassette	5′-CGCTCACATTGGTACCGGTTCC-3′
PKACpN5-R	Reverse primer to amplify 695 bp 5′ region of *CgPKAC* for construction into hygromycin cassette	5′-ACGGAAGGATCCAGGGCAATCA-3′
PKACpN3-F	Forward primer to amplify 376 bp 5′ region of *CgPKAC* for construction into hygromycin cassette	5′-ACGGGCATGCGGCCGCGGTTTCT-3′
PKACpN3-R	Reverse primer to amplify 376 bp 5′ region of *CgPKAC* for construction into hygromycin cassette	5′-CCTTTGAAGCGCATGCCCGACCC-3′
